# Self-Powered Wearable Pressure Sensors with Enhanced Piezoelectric Properties of Aligned P(VDF-TrFE)/MWCNT Composites for Monitoring Human Physiological and Muscle Motion Signs

**DOI:** 10.3390/nano8121021

**Published:** 2018-12-07

**Authors:** Aochen Wang, Ming Hu, Liwei Zhou, Xiaoyong Qiang

**Affiliations:** School of Microelectronics, Tianjin University, Tianjin 300072, China; 13602176911@163.com (L.Z.); shawn_q@tju.edu.cn (X.Q.)

**Keywords:** self-powered electronics, piezoelectric pressure sensor, P(VDF-TrFE), MWCNTs, electrospinning, wearable biomedical devices

## Abstract

Self-powered operation, flexibility, excellent mechanical properties, and ultra-high sensitivity are highly desired properties for pressure sensors in human health monitoring and anthropomorphic robotic systems. Piezoelectric pressure sensors, with enhanced electromechanical performance to effectively distinguish multiple mechanical stimuli (including pressing, stretching, bending, and twisting), have attracted interest to precisely acquire the weak signals of the human body. In this work, we prepared a poly(vinylidene fluoride-trifluoroethylene)/ multi-walled carbon nanotube (P(VDF-TrFE)/MWCNT) composite by an electrospinning process and stretched it to achieve alignment of the polymer chains. The composite membrane demonstrated excellent piezoelectricy, favorable mechanical strength, and high sensitivity. The piezoelectric coefficient d_33_ value was approximately 50 pm/V, the Young’s modulus was ~0.986 GPa, and the sensitivity was ~540 mV/N. The resulting composite membrane was employed as a piezoelectric pressure sensor to monitor small physiological signals including pulse, breath, and small motions of muscle and joints such as swallowing, chewing, and finger and wrist movements. Moderate doping with carbon nanotubes had a positive impact on the formation of the β phase of the piezoelectric device, and the piezoelectric pressure sensor has the potential for application in health care systems and smart wearable devices.

## 1. Introduction

Flexible and wearable pressure sensors for human health monitoring, disease diagnosis, artificial skin, and biomedical prosthesis have attracted tremendous attention [1,2,3,4,5,6,7,8,9]. The flexibility of pressure sensors is important to ensure the device closely contacts the human skin or organ, which is beneficial to the acquisition and measurement of biological signals. The fabrication of pressure sensors is mainly based on transduction modes including resistivity [10,11], capacitivity [12,13], piezoelectricity [14,15], and triboelectricity [16,17]. Compared with non-piezoelectric pressure sensors that need an extra power supply to function normally, piezoelectric pressure sensors are self-powered, portable, and secure, which makes them competitive in sensing applications [18].

Piezoelectric polymers can be easily deformed to generate electric responses by different actions such as bending, stretching, and pressing. This mechanism of action depends on the piezoelectric effect. When a piezoelectric polymer is subjected to physical force, the dipole moment in the material shortens due to the compression. To counter this change, the piezoelectric material produces an equal amount of positive and negative charges on the relative surface in order to maintain the original state. Combined with other advantages, including the controllability of its nanostructures, easy preparation, good chemical stability, and biocompatibility, piezoelectric polymers are considered promising candidate materials for the fabrication of piezoelectric sensors. However, under minimal mechanical force, piezoelectric sensors output weak electrical signals, which are mainly limited by the piezoelectric properties of polymer. Poly(vinylidene fluoride) (PVDF) and its copolymer poly(vinylidene fluoride-trifluoroethylene) (P(VDF-TrFE)) are representative piezoelectric materials due to their outstanding and unique piezoelectric property given their β piezoelectric crystallization phase [19]. If the dipoles in the β phase are entirely oriented in the same direction, the polymer generates the largest piezoelectric outputs [20]. Nevertheless, it is difficult to completely convert the non-polar phases to the β polar phase. In order to achieve the β crystallization phase, a mechanical drawing method can be used to force the molecules to rotate into the TTTT chain conformation known as the β polar phase. Mechanical drawing is also used to align the stacking molecular chain along the force direction [21,22]. Sun et al. [23] found that after mechanical drawing, carbon nanofibers (CNF) (greater than 0.2 wt %)/PVDF composites obtained a remarkably high β phase (~80%). The electrospinning process that can supply local electrical poling and preliminary drawing force has attracted interest for fabricating nano-structure fibers [24,25,26].

Many studies have demonstrated that carbon materials (such as carbon nanotubes or fibers and graphene) can be used as nanofillers to adjust the structure and promote the formation of the polar β phase, thus improving the piezoelectricity of polymers [27,28,29]. Based on the density functional theory, the molecular chains with TTTT conformation are more tightly bound to the surface of carbon nanotube than the chains with α phase conformation. Levi et al. [30] found that P(VDF-TrFE)/single-walled carbon nanotubes (SWCNTs) (d_31_ coefficient was 25 pc/N) prepared by solution casting exhibited a higher piezoelectric coefficient than pure P(VDF-TrFE) film (d_31_ was 20 pc/N). Chen et al. [31] prepared polyethylene glycol grafted graphene (PEG-graphene) and PVDF composites, and found that the electroactive crystallinity of PVDF was significantly improved through the interfacial interaction with PEG-graphene. The addition of CNTs can improve the sensitivity and mechanical properties of a pressure sensor [32,33,34,35].

In this study, we fabricated P(VDF-TrFE)/multi-walled carbon nanotube (MWCNT) composites by an electrospinning process. After treatment by a mechanical drawing method, the composite membrane possessed a high β polar phase content, high sensitivity, and favorable mechanical robustness. We processed the composite membrane as a piezoelectric pressure sensor, and then explored its sensing performance in practical application scenarios for monitoring human physiological signs, including pulse and breathing, muscle and joints motion signals, as well as the ability to distinguish different models of joint movements. The experimental results indicate that the enhanced piezoelectric pressure sensor shows potential application in personal health care and disease diagnosis.

## 2. Experimental Section

### 2.1. Materials

We purchased the polymer P(VDF-TrFE) powder (with a molar ratio of 75/25, Piezotech Inc., Pierre-Bénite, France), MWCNTs (≥95%, average diameter of 5–10 nm, length of 10–30 µm, Aladdin, Shanghai, China), *N*,*N*-dimethylformamide (DMF, 99.9%, Sigma Aldrich, Saint Louis, MO, USA), acetone (AR, 99.5%, Sigma Aldrich, USA) and silicone rubber (volume ratio of its base and cure was 1:1, Ecoflex 00-30, Smooth-on Inc., Macungie, PA, USA).

### 2.2. Preparation of Aligned P(VDF-TrFE)/MWCNT Composite Membranes

The 0.2 wt % MWCNTs were dispersed in DMF solvent by ultrasonic bath for 30 min to ensure uniform dispersion of the nanotubes. The 20 wt % P(VDF-TrFE) powder was dissolved in a mixed solution of MWCNT solvent and acetone with a volume ratio of 6:4 by magnetic stirring for 4 h. The polymer solution was then injected in a 5 mL commercial plastic syringe tipped with a 22-gauge steel needle. The electrospinning process was performed at a high voltage of 15 kV. The collection distance between the needle tip and dish collector with a rotating speed of 1800 rpm was set to 10 cm. The solution was fed into the needle tip at the rate of 1 mL·h^−1^ with a syringe pump (KDS101, KD Scientific, Holliston, MA, USA). All electrospun membranes were dried at a constant temperature of 65 °C in an oven for 24 h.

### 2.3. Fabrication of the Piezoelectric Pressure Sensor

First, the electrospun membranes were placed on a uniaxial stretching machine, followed by drawing treatment with a stretching rate of 0.2 mm·s^−1^ at 115 °C. The samples were elongated to two times the original length. After that, the samples were annealed at 135 °C in a vacuum oven for 4 h and then cut into 1.5 cm× 1.5 cm pieces with thickness of ~30 µm. Electrodes with an area of 1.2 cm × 1.2 cm sputtered with Au were placed on the top and bottom of the membranes, linking with copper wires for the following test. Finally, the devices were encapsulated with silicone rubber. The total thickness of the device was ~2 mm. In the subsequent section, the electrospun pure P(VDF-TrFE) after mechanical drawing treatment is labeled as stretched P(VDF-TrFE) and the composite membrane with mechanical drawing treatment is labeled as stretched P(VDF-TrFE)/MWCNTs.

### 2.4. Characterization and Measurements

The sample morphology was examined by scanning electron microscopy (SEM, SU8020, Hitachi Ltd., Tokyo, Japan). X-ray diffraction (XRD) patterns of the samples were recorded with a X-ray diffractometer (X′pert3, PANalytical Ltd., Almelo, The Netherlands) with a step size of 0.013° at a scanning 2θ range of 10–50°. XRD was operated with a CuKα source at 40 kV and 40 mA. Fourier transformed infrared spectroscopy (FTIR) spectra were collected Using a Vertex80V (Bruker Corp., Billerica, MA, USA) in the range of 400–1600 cm^−1^ with a resolution of 4 cm^−1^. Differential scanning calorimetry (DSC) curves were measured by a DSC instrument (TGA/DSC1, Mettler-Toledo LLC., Columbus, OH, USA) at a heating/cooling rate of 10 °C/min in the range of 40 to 180 °C in an argon environment. Polarization-electric field (P-E) hysteresis loops were measured by a precision multiferroic and ferroelectric tester (Radiant Technologies Inc., Alpharetta, GA, USA) with unipolar electric field at a frequency of 10 Hz. The Young’s modulus of the samples was obtained using a dynamic mechanical analysis (DMA) system (Q800, TA Instrument, New Castle, DE, USA). The tensile test was performed using a tensile test machine (ESM301, Mark-10, Copiague, NY, USA). A dumbbell-shaped sample with a gauge length of ~10 mm and a width of ~5 mm was prepared and tested at room temperature under a crosshead speed of 10 mm/min. The piezoelectric response was measured via piezoresponse force microscopy (PFM) by applying a bias voltage on a Pt/Ir-coated silicon tip with a force constant of 2.8 N/m in the range of −20 to 20 V. The output electric signals of the sensors were recorded by an electrometer (6514, Tektronix Keithley Inc., Beaverton, OR, USA) and depicted through LabView software (2016, National Instruments, Austin, TX, USA).

## 3. Results and Discussion

**Figure 1:**
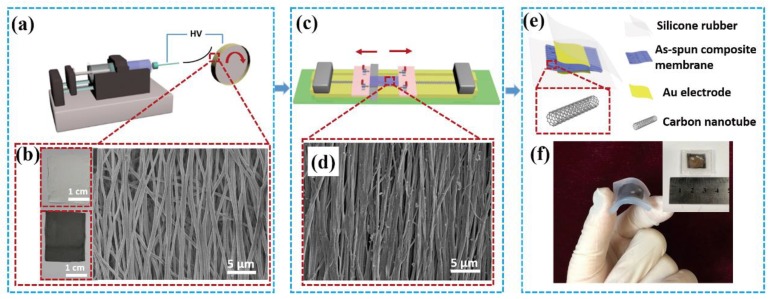
Overview of the piezoelectric pressure sensor. (**a**) Schematic diagram showing the experimental setup for fabrication of PVDF-TrFE/MWCNTs nanofibers. (**b**) Image of electrospun PVDF-TrFE (upper left) and PVDF-TrFE/MWCNTs nanofibers (lower left) and SEM micrograph of the electrospun composites nanofibers (right). (**c**) Schematic diagram of the mechanical drawing experimental setup. (**d**) SEM micrograph of the stretched composites nanofibers. (**e**) Schematic drawings showing the structure and flexibility of the sensor. (**f**) A flexible sensor sample and inset showing the front of the sensor.

A schematic diagram of the preparation of the P(VDF-TrFE)/MWCNT composites membranes via the electrospinning process is illustrated in Figure 1a. The electrospun nanofibers were preliminarily stretched through the high-speed rotating collecting disk, forming an aligned nanofiber mat (Figure 1b). Figure 1c shows the aligned mat was further stretched through the mechanical drawing treatment. Under the external mechanical force, the molecular chains of the polymer oriented in the direction of the force and then the chains were arranged in an orderly manner. The nano-morphology of the stretched composite nanofibers is demonstrated in the SEM image in Figure 1d. The electrospun composites nanofibers were bead-free with highly-aligned topography as well as a porosity conducive to the sensitivity of the pressure sensor [36,37]. Figure 1e exhibits the fundamental structure of the piezoelectric pressure sensor. The top and bottom of the composite membrane were covered with Au electrodes to guarantee good conductivity. The membrane was then encapsulated with translucent silicone rubber layers to ensure the mechanical durability of the pressure sensor and isolation from interference from the ambient environment. The resulting pressure sensor exhibited in Figure 1f showed remarkable flexibility. The device was fabricated using a low-cost and simple method, with its shape and size easily adjusted to meet various needs.

**Figure 2:**
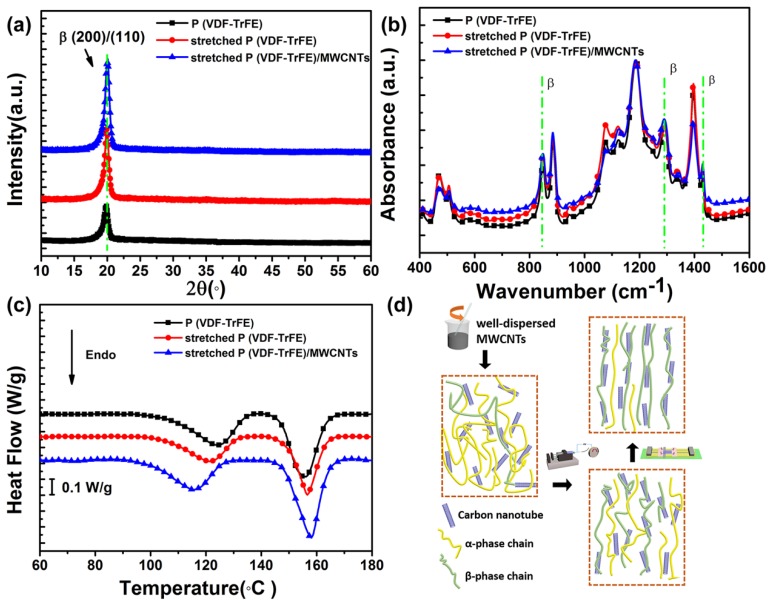
The crystalline and thermal characterization of P(VDF-TrFE)/MWCNT composites membranes. (**a**) XRD patterns of different prepared samples. (**b**) FTIR spectra. (**c**) DSC curves. (**d**) Schematic of the proposed mechanism of different processes and the effect on the formation of the β phase.

To investigate the influence of mechanical drawing and MWCNTs doping treatments on the formation of the piezoelectric β-phase, the crystal structures of various samples were characterized by XRD patterns, FTIR spectra, and DSC curves. The characteristic peaks of the β polar phase in the XRD patterns shown in Figure 2a are near 20°, which corresponds to the (200)/(110) reflection [38]. The diffraction intensity of the β crystalline peaks at 19.9° increased significantly after drawing treatment, which indicates mechanical stretching promotes the generation of the piezoelectric β phase. Under the combined action of mechanical drawing and MWCNT doping treatments, the intensity of the diffraction peak significantly increased. By fitting the patterns of the samples [39], the curve could be divided into α and β phases; thus, their quantification could be evaluated. The percentage of the piezoelectric β phase in stretched P(VDF-TrFE)/MWCNT was 85.5%, whereas that of the stretched P(VDF-TrFE) membrane was 72.1% compared to the 59.9% of the original P(VDF-TrFE) membrane (Figure S1).

The FTIR spectra of the samples are presented in Figure 2b. The main characteristic absorption peaks associated with the β phase appeared at 848, 1285, and 1340 cm^−1^ [40], and corresponding peaks associated with the α phase occurred at 765, 870, 976, and 1211 cm^−1^ [41]. The intensity of the absorption peaks associated with the β phase increased gradually with drawing treatment and MWCNTs doping, which is consistent with the XRD patterns.

DSC measurement was also employed to investigate the crystal phase structure, as shown in Figure 2c. The different ferroelectric phases, including α, β, and γ, possess different thermodynamic stability. The Curie transition peak temperatures of the ferroelectric phase above 118 °C correlate to the α phase, whereas the temperatures of the β phase range from 110 to 118 °C [42]. The Curie transition peak of the stretched sample narrowed and shifted to a lower temperature than the non-stretched sample, indicating the transition of the ferroelectric phase from a stable α phase to a less stable β phase. The doping of MWCNTs, the increase in the Curie transform enthalpy, the sharper melting peak, and the upward movement of the melting temperature all confirm an increase in the β phase, suggesting that MWCNT doping can enhance the β phase transition.

The fraction of the β phase in the piezoelectric crystals mainly depends on the dispersion of nanofillers and interfacial interactions between the nanofillers and polymer matrix, as well as post treatments such as annealing, stretching, and poling. As shown in Figure 2d, the homogeneous dispersion of MWCNTs in the polymer matrix enhanced the wrapping of molecular chains on the surface of carbon nanotubes, increased interfacial interactions, and promoted the nucleation of the β phase of P(VDF-TrFE) [43]. The mechanical drawing treatment facilitated the orientation of the polymer chains along the direction of the force, which further enhanced the interaction between the polymer matrix and MWCNTs. Accordingly, the combined effects of doping of MWCNTs and the drawing treatment are favorable to the formation of the β phase [44].

**Figure 3:**
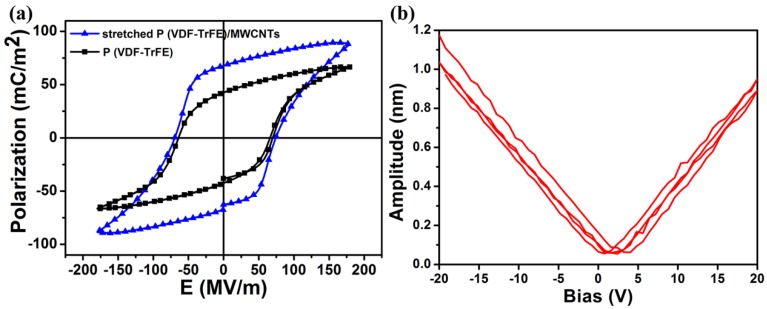
The piezoelectric performance of the stretched P(VDF-TrFE)/MWCNT membrane. (**a**) P-E hysteresis loops. (**b**) PFM amplitude-bias hysteresis loops.

Representative tensile stress-strain curves of the three nanofiber membranes are shown in Figure S2. The average elastic modulus of the original P(VDF-TrFE), stretched P(VDF-TrFE), and stretched P(VDF-TrFE)/MWCNT were 0.197 GPa, 0.588 GPa, and 0.990 GPa, respectively. Using DMA, the Young’s modulus of the prepared sensor was ~0.986 GPa, which is consistent with the tensile test results. This significant enhancement is attributed to the mechanical drawing treatment, which makes the arrangement of fibers more ordered, better distributing the tensile stress. After doping with MWCNTs, the Young’s modulus increased by 503% compared to the original P(VDF-TrFE) membrane, which was mainly due to the doping with the high toughness MWCNT that interacted well with the P(VDF-TrFE) matrix. The stretched P(VDF-TrFE) membrane exhibited much lower toughness than the other two membranes. The reduction in toughness was due to the low mobility of the molecular chain in the crystalline phase that reduces stretching at room temperature compared to the amorphous phase. These results indicate that the MWCNTs incorporation significantly enhanced the mechanical strength of the membrane, which is important for practical applications. The robust mechanical property of the piezoelectric pressure sensor also lays the foundation for its piezoelectric output performance [45]. The effects of mechanical drawing and MWCNT doping treatments on the P-E hysteresis loops are exhibited in Figure 3a. The stretched P(VDF-TrFE)/MWCNT membrane showed a more saturated hysteresis loop than the non-stretched P(VDF-TrFE) membrane. The remnant polarization (Pr) value of the stretched P(VDF-TrFE)/MWCNT sample was 67.7 mC/m^2^ at 179 MV/m, whereas that of the non-stretched P(VDF-TrFE) sample was 42.4 mC/m^2^, indicating significant enhancement of the ferroelectric properties with the addition of MWCNTs and mechanical drawing treatment. The longitudinal piezoelectric coefficient d_33_ is approximately 50 pm/V according to the slope of the displacement and bias voltage curves from the PFM morphology presented in Figure 3b, indicating the stretched P(VDF-TrFE)/MWCNT membrane showed good piezoelectricity. Accordingly, a stretched P(VDF-TrFE)/MWCNT-membrane-based piezoelectric pressure sensor offers a nimble and promising approach for wearable devices and energy harvesting applications.

**Figure 4:**
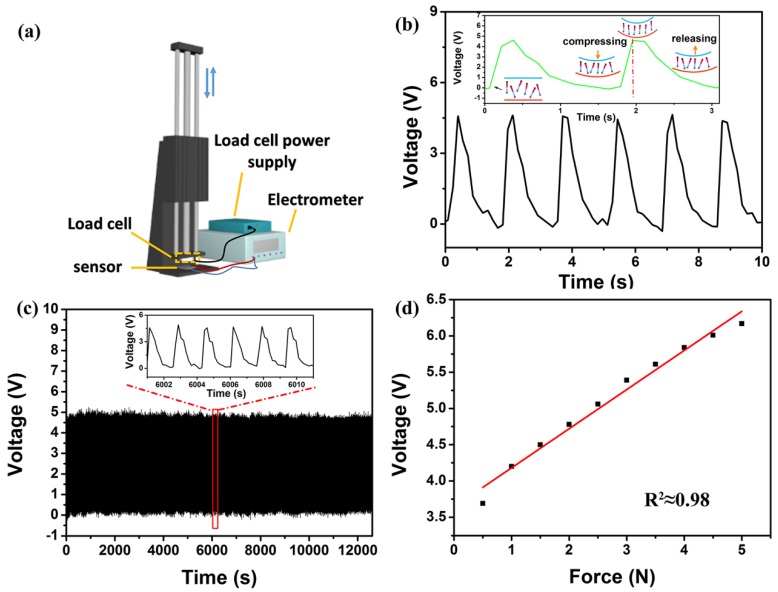
The sensing performance of the piezoelectric pressure sensor based on stretched P(VDF-TrFE)/MWCNT composites. (**a**) Schematic diagram of the designed experimental setup. (**b**) The output voltage response to the external force of 2.5 N with a frequency of 0.6 Hz. The inset shows working mechanism of the piezoelectric pressure sensor. (**c**) The stability measurement over 3.5 h under a 2.5 N stress. The inset shows a magnified 10 cycles. (**d**) The linear relationship between generated voltage and the compressive force in the range of 0.5 to 5.0 N.

To evaluate the sensing performance (including stability and sensitivity) of the piezoelectric pressure device under vertical mechanical force, an experimental setup was designed to supply periodical compression. The schematic diagram of the experimental setup is depicted in Figure 4a. A linear motor was used to apply an accurate and periodic compressive force that was measured by the load cell. The output voltage of the prepared pressure sensor was 5 V when a vertical compressive force of 2.5 N with a frequency of 0.6 Hz (Figure 4b) was exerted. The inset of Figure 4b illustrates the working mechanism of the piezoelectric pressure sensor. In brief, when the linear motor came into contact with the surface of the device, the device was not deformed, so the output voltage was 0 V. Then, the linear motor was continuously compressed downward, the deformation of the device increased, and the output voltage increased accordingly. Finally, the deformation reached a maximum and the output voltage reached a peak value. As the linear motor gradually separated from the device, the deformation gradually recovered. The equal force was applied over 3.5 h, and the results shown in Figure 4c display that there was no obvious voltage degradation, revealing that the prepared pressure sensor possesses favorable mechanical stability. In addition, the output voltage varied linearly as a function of forces ranging from 0.5 N to 5 N, as shown in Figure 4d. The piezoelectric sensitivity of the pressure sensor was approximately 540 mV/N according to the slope of the linear curve, which was superior to previously reported piezoelectric pressure sensors [46,47]. Table 1 summarizes the piezoelectric coefficient d_33_ value, Young’s modulus, and sensitivity of the PVDF-based membranes in our study compared to literature values. The combination of the drawing treatment and MWCNT doping was beneficial to enhancing the sensitivity and piezoelectric and mechanical properties of the pressure sensor.

**Figure 5:**
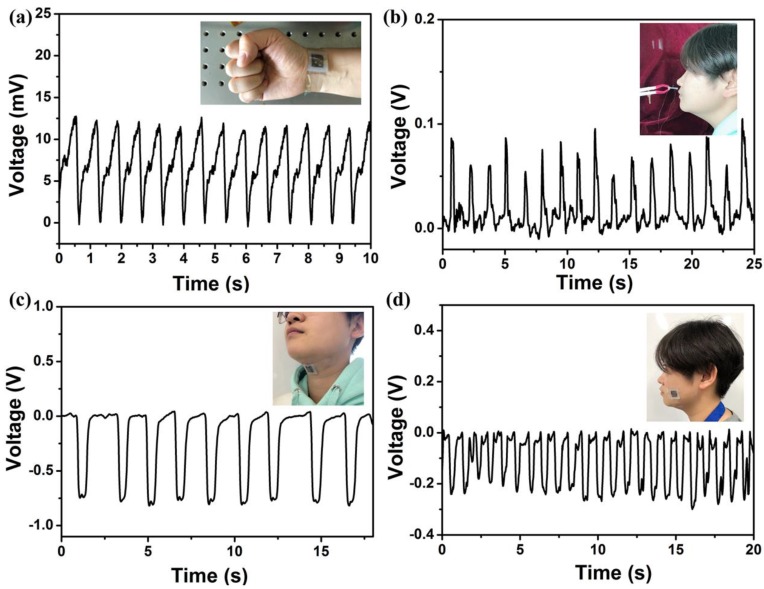
The piezoelectric pressure sensor used for monitoring vital signs and muscle motion. The voltage output of detected (**a**) pulse rate, (**b**) breathing, (**c**) swallowing, and (**d**) chewing. The inset photographs represent the position where the sensor was mounted.

Based on the measurements of the pressure sensor properties, a series of experiments were conducted to demonstrate practical application in human health monitoring. The prepared piezoelectric pressure sensor was first mounted on the wrist to detect and record the blood pulse signal. The achieved real-time output signal of an adult pulse is shown in Figure 5a. The recorded pulse rate was approximately 84 beats per minute, which is normal, demonstrating the sensor possesses superb sensitivity to obtain a real-time response to a relatively weak signal. The sensor was then used to detect human breathing by clamping to eliminate interference from finger motion. The corresponding output voltage is shown in Figure 5b, where the peak value was approximately 0.1 V. When the subject exhaled, the sensor was bent and deformed by the gentle airflow, and the output voltage changed in response to the depth of respiration. As shown in Figure 5c, the sensor was attached above the throat to measure the electric signals of the swallowing behavior; the peak output voltage was approximately 0.75 V. A flat region also appeared in the wave peaks and wave troughs which may have been the result of the time for hyolaryngeal muscles movement [48]. This result indicates that the sensors can be used as a feedback treatment for chronic dysphagia. The sensor was also employed to detect the signals of the masticatory movement. The electric output under continuous chewing activities is shown in Figure 5d, where the peak voltage was 0.3 V. These results prove that the prepared piezoelectric pressure sensor is appropriate for monitoring vital signs and muscular movements of human beings.

**Figure 6:**
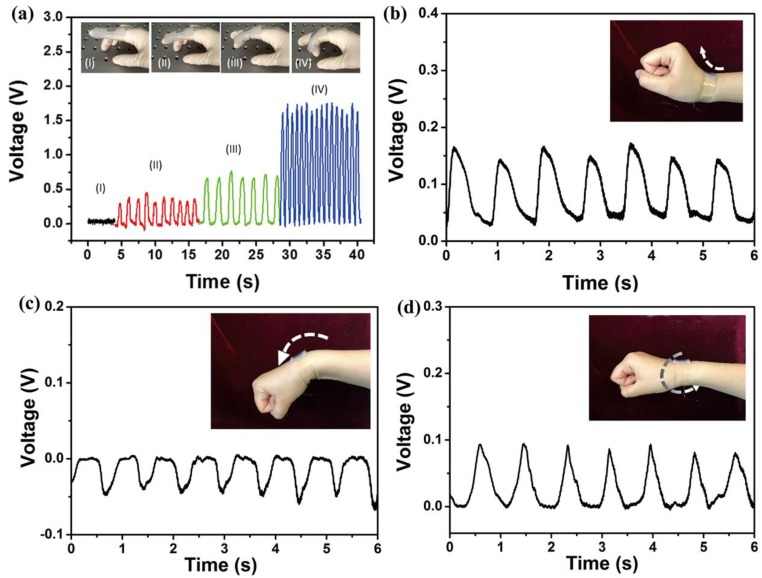
The piezoelectric pressure sensor used for detecting joint motion states. (**a**) The sensor used for detecting finger motion states. Change in generated voltage under different types of wrist movements: (**b**) upward bending, (**c**) downward bending, and (**d**) torsion.

A group of joint motion tests were also conducted to examine the performance of the piezoelectric pressure sensor. First, the sensor was placed on the knuckle, and the output voltage response was proportional to the degree of bending of the finger, and the voltage reached a maximum of approximately 1.8 V, as shown in Figure 6a. The sensor was fixed on the wrist to distinguish different kinds of wrist movements, including upward bending, downward bending, and torsion, as shown in Figure 6b–d, respectively. When the wrist moved upward and downward, the sensor bent accordingly, and the corresponding maximum and minimum output voltage was approximately 0.15 V and −0.05 V, respectively. The difference in the output voltage might be due to the difference in sensor deformation. When the wrist was twisted, the sensor was subjected to the combined force of upward, shear, and downward forces, so the output voltage was the constructive or destructive combination of the corresponding response. These results indicate that the prepared piezoelectric pressure device based on the stretched P(VDF-TrFE)/MWCNT composite membrane not only has stretchable and wearable features, but can also output different responses that are associated with different kinds of human motions, demonstrating its versatility and potential for practical applications.

## 4. Conclusions

In this paper, we introduced a piezoelectric pressure sensor based on a P(VDF-TrFE)/MWCNT composite membrane created via an electrospinning process and mechanical drawing treatment. A series of measurements, including XRD patterns, FTIR spectra, DSC, and P-E loops, were conducted to characterize the piezoelectric properties of the composite membrane. The results showed that the acceleration of the formation of the β phase, and thus the enhancement of the piezoelectricity, is the result of the combined effect of MWCNTs and mechanical drawing treatment. A small amount of MWCNTs promote the nucleation of the β phase of P(VDF-TrFE), and mechanical drawing treatment enhances the interaction between the polymer matrix and MWCNTs due to the alignment of the polymer chains. An experimental setup was used to investigate the sensing performance and mechanical property via the Young’s modulus. The results indicated that the prepared piezoelectric pressure sensors possess excellent sensitivity, robust mechanical flexibility, long-term mechanical stability, and good linearity to vertical compressive forces. Finally, a group of tests of physiological signs and muscle and joint motions were conducted to examine the performance of the sensor in practical application. The results revealed the sensor is stretchable and wearable, and can differentiate multiple kinds of human motions. In summary, the enhanced piezoelectricity, excellent sensitivity, robust mechanical flexibility and other merits make the piezoelectric pressure sensor suitable for health care systems, smart wearable devices, and in vitro disease diagnosis.

## Figures and Tables

**Table 1:** Summary of the characteristics of the PVDF-based membranes in this study and other recently reported studies.

Material	Form	d_33_ (pm/V)	Young’s Modulus (GPa)	Sensitivity (mV/N)	Ref.
CNT-PVDF	Thin film	13	-	-	[49]
P(VDF-TrFE)/SWCNTs	Film	25	-	-	[30]
PVDF/CNT	Nanofiber	31.3	-	2.26	[50]
P (VDF-TrFE)	Nanowire	-	-	458.2	[46]
P(VDF-TrFE)/SWCNTs	Film	-	0.908	-	[51]

## Supplementary Materials

The following are available online at http://www.mdpi.com/2079-4991/8/12/1021/s1, Figure S1: The fitting results of XRD patterns. Figure S2: Tensile test curves of different samples.
