# Supplementary material for: Self-Powered Wearable Pressure Sensors with Enhanced Piezoelectric Properties of Aligned P(VDF-TrFE)/MWCNT Composites for Monitoring Human Physiological and Muscle Motion Signs
Source: Nanomaterials (Basel). 2018 Dec 7;8(12):1021. doi: 10.3390/nano8121021 (PMC6315454; doi:10.3390/nano8121021)
Supplement: Supplementary file 1 [file nanomaterials-08-01021-s001.pdf]

nanomaterials

MDPI

Self-powered Wearable Pressure Sensors with Enhanced Piezoelectric Properties of Aligned P(VDF-TrFE)/MWCNT Composites for Monitoring Human Physiological and Muscle Motion Signs

Aochen Wang *, Ming Hu *, Liwei Zhou, and Xiaoyong Qiang

School of Microelectronics, Tianjin University, Tianjin 300072, China; 13602176911@163.com (L.Z.); shawn_q@tju.edu.cn (X.Q.)

* Correspondence: aochen_wang@tju.edu.cn (A.W.); huming@tju.edu.cn (M.H.)

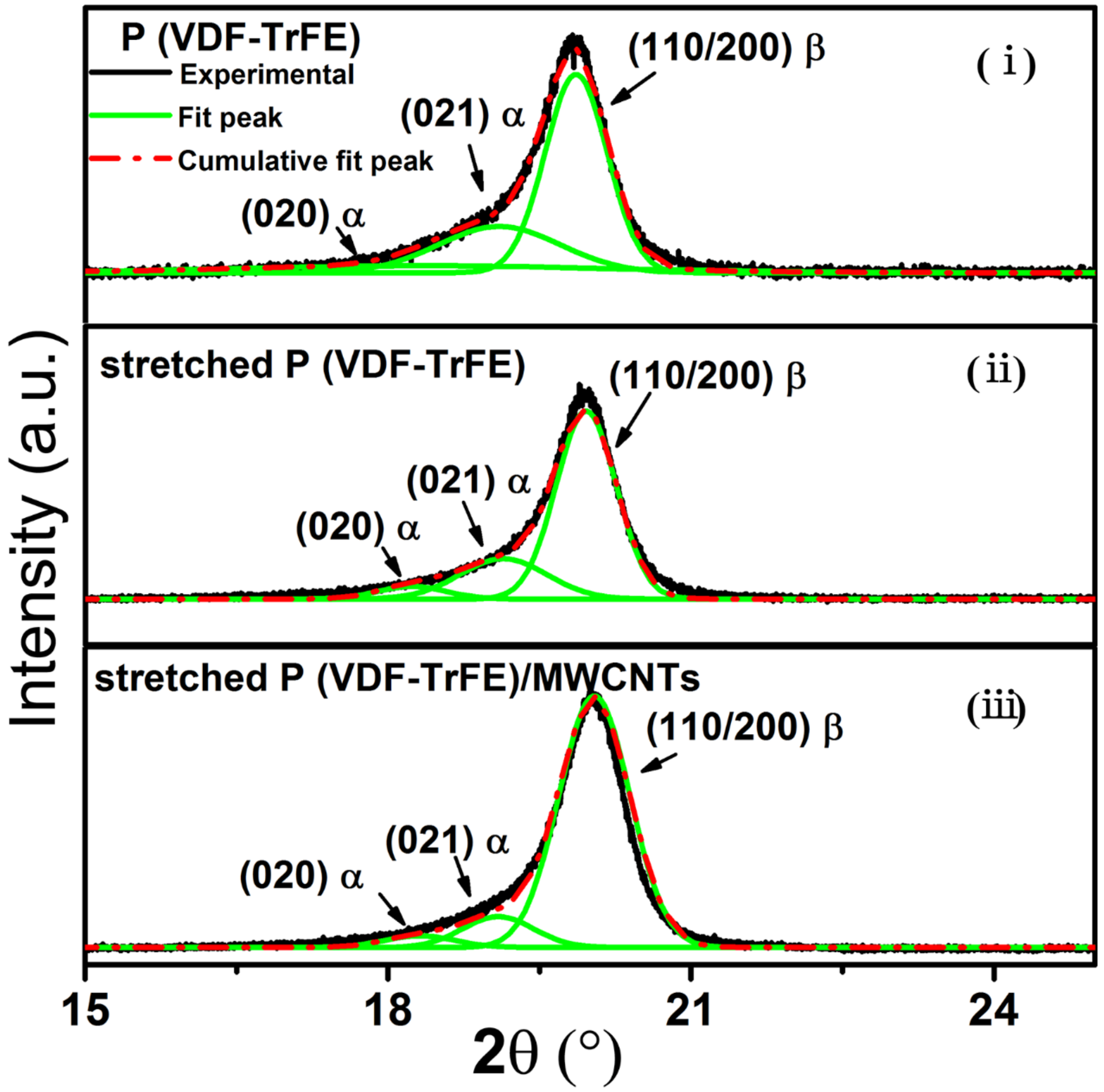

Figure S1. The fitting of XRD patterns of different samples: (**i**) Original P(VDF-TrFE) membrane, (**ii**) stretched P(VDF-TrFE) membrane, and (**iii**) stretched P(VDF-TrFE)/MWCNT composites membrane (from top to bottom).

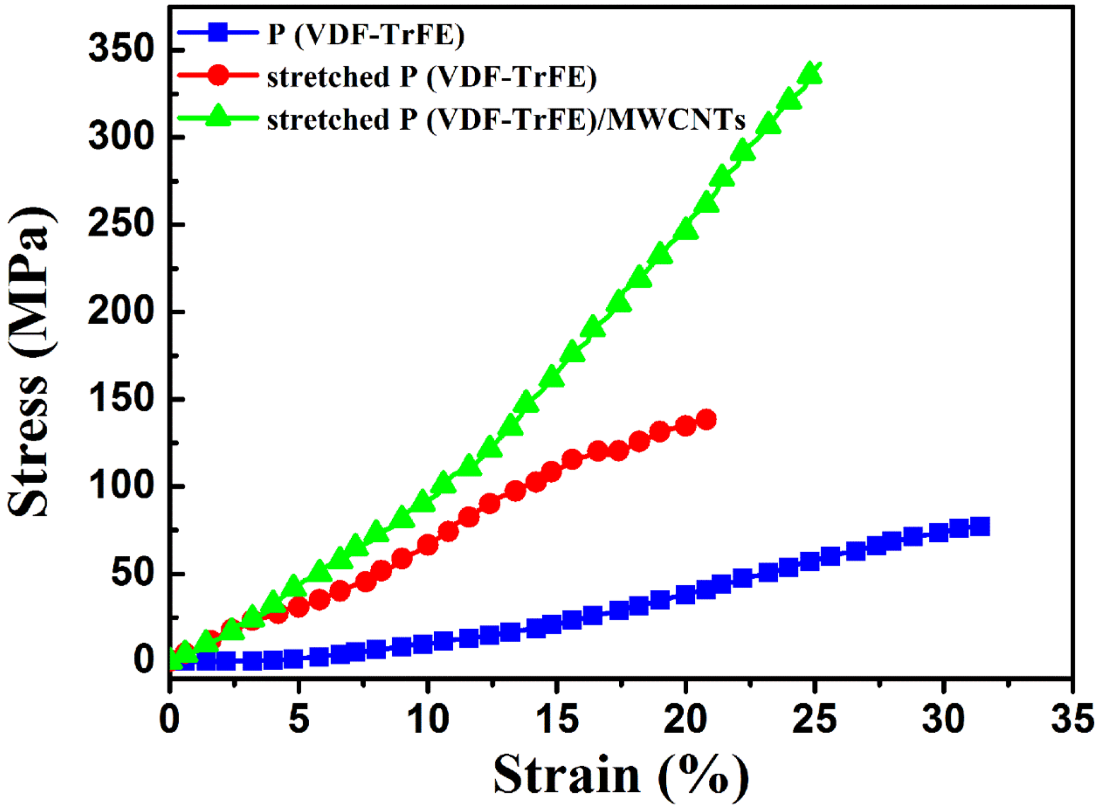

Figure S2. Tensile test curves of different samples.
